# Supplementary material for: Identification of photoperiod-induced specific miRNAs in the adrenal glands of Sunite sheep (Ovis aries)
Source: Front Vet Sci. 2022 Jul 22;9:888207. doi: 10.3389/fvets.2022.888207 (PMC9354845; doi:10.3389/fvets.2022.888207)
Supplement: Supplementary file 1 [file Data_Sheet_1.ZIP › Supplementary file/miRDeep2 file.zip!/miRDeep2 file/Know_Structure/LP42A1/oar-mir-539.pdf]

miRBase precursor : oar-mir-539
Total read count : 927
oar-miR-539-5p read count : 5
oar-miR-539-3p read count : 922
remaining reads : 0

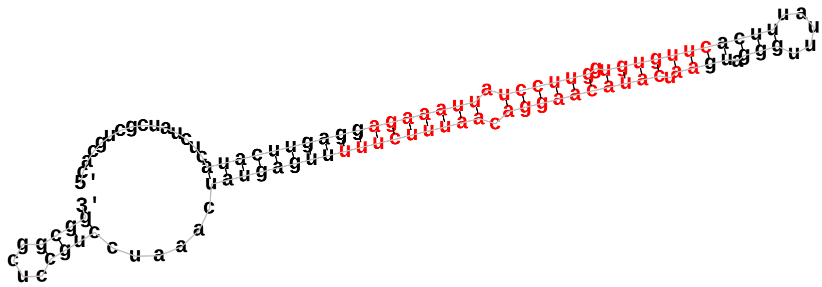

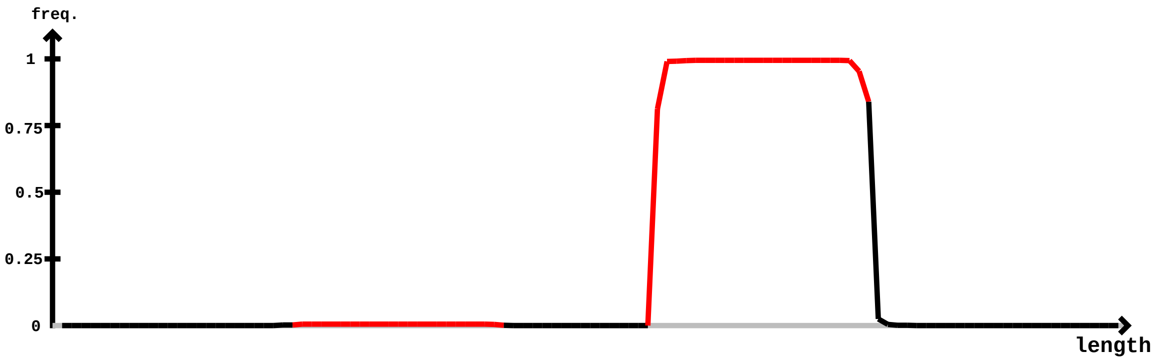


```
                                                                oar-miR-539-3p
                         oar-miR-539-5p
5'- cacgucgcuaucucauacuugaggagaaauuauccuugguguguucacuuuauuugggaugaaucauacaaggacaauuucuuuuugaguaucaaauccugccucggcgg  -3'  exp
    ..............(((((((((((((((((.((((((.(((((((((((.....))).)))).)))))))))).)))))))))))))))))......((((....))))  reads   mm   sample
    ......................ggagaaauuauccuuggugugu..................................................................  1       0    seq
    ......................ggagaaauuauccuuggugugugu.................................................................  1       0    seq
    ........................agaaauuauccuugguguguu.................................................................  2       0    seq
    ........................agaaauuauccuugguguguuc................................................................  1       0    seq
    .............................................................aaucauacaaggacaauuuc.............................  1       0    seq
    .............................................................aaucauacaaggacaauuucu............................  37      0    seq
    .............................................................aaucauacaaggacaauuucuu...........................  103     0    seq
    .............................................................aaucauacaaggacaauuucuuu..........................  604     0    seq
    .............................................................aaucauacaaggacaauuucuuuu.........................  8       0    seq
    ..............................................................aucauacaaggacaauuucuu...........................  3       0    seq
    ..............................................................aucauacaaggacaauuucuuu..........................  151     0    seq
    ..............................................................aucauacaaggacaauuucuuuu.........................  11      0    seq
    ...............................................................ucauacaaggacaauuucuuuu.........................  1       0    seq
    ................................................................cauacaaggacaauuucuuuu.........................  2       0    seq
    .................................................................auacaaggacaauuucuuuuuga......................  1       0    seq
```
